# Career perspective: Kenneth J. Collins

**DOI:** 10.1186/s13728-017-0058-4

**Published:** 2018-01-23

**Authors:** Kenneth John Collins

**Affiliations:** London, UK

**Keywords:** Temperature regulation, Endocrine control, Working capacity, Tropical medicine, Heat stroke, Hypothermia, Autonomic failure, Seasonal mortality

## Abstract

A career interest in thermoregulation research has included wide contrasts in the subjects of enquiry, extending from heat stroke to hypothermia, special investigations in many different purpose-built climatic chambers, laboratory-based biomedical studies together with hospital practice, and field work in tropical climates to physiological surveys on urban populations in temperate environments. The scientific process and need to focus on careful planning of experiments, using the most appropriate methods, selecting the right controls and eventually applying correct statistical analysis do not always follow a smooth transition, as illustrated in this account. The result of endeavour to resolve a human environmental problem, however, is greatly satisfying, and sometimes becomes a unique experience when the solution reveals new fundamental facts.

## Background

It was a privilege to have been able to study physiology in G. L. Brown’s Department at University College London (UCL) in the early 1950s. The names on the doors of offices and laboratories along the central corridor were all of eminent physiologists—A. V. Hill, L. E. Bayliss, H. Davson, M. de B. Daly, A. Schweitzer, B. Katz, and J. A. B. Gray. The imposing main lecture theatre which has existed, since Starling’s day has been a central venue for many notable Physiological Society meetings. With such a Departmental staff on site, the theatre meetings were always compulsive with lively exchanges. During the course of subsequent studies in physiology and medicine, I have found that the Physiological Society has consistently provided a supporting high standard on which to base a career.

Within a few weeks of graduation at UCL in 1954, I moved to Oxford to join a research group led by J. S. Weiner, the MRC climate and working efficiency unit, situated in Wilfrid Le Gros Clark’s Department of Anatomy. Weiner was a dynamic leader, well known as an anthropologist who had just completed writing his seminal book exposing the Piltdown man forgery. I soon found that the attraction of an MRC research post meant being obliged to stand scientifically on ones’ own feet, to find my own problems, to plan experiments, and to devise methods for myself. A number of experimental projects were centred on the Unit’s climatic chamber which had recently been constructed to contain a treadmill with transparent side walls for accurate air movement control. We did not have the benefit of computers at the time and the Fortran programming language was only just about to be introduced. There was some indecision about computers; Lewis Thomas wrote ‘With computers we can leap over mountains of data, and land lightly on the wrong side’. Statistical analysis relied entirely on the calculating machine. On reflection, however, it is remarkable that the Unit combined studies with the same juxtaposition of exercise physiology and the environment that still applies today, including members specialising in applied psychology as well as the emerging science of ergonomics through the work of J. S. Whitney who was engaged in testing an early force analysis platform and he had personally constructed.

It was an integral part of the Unit’s remit to undertake research on ad hoc problems requested by Government and industry which were received by the MRC. One of the major projects involving members of the Unit’s staff was a National Coal Board investigation into the reactions of mines-rescue personnel working in severely hot conditions. Permissible lengths of time for which men could engage in rescue operations in such conditions were studied both in the laboratory and at the coal face. I took part in several expeditions to deep, hot mines in the UK and recall that knowledge on how to survive in the hazardous surroundings underground had to be acquired very quickly.

## Thermoregulation and survival in hot environments

The main study in a broad spectrum of climate research which had been stimulated by war-time experiences in the field focused on the need to establish suitable empirical heat-stress indices with which to predict safe limits for working practices in hot climates. We continued this research on larger numbers of personnel in the more capacious climate chambers of the Services Institutes at Farnborough. In the extreme environments, we employed that it was necessary to wear ventilated protective suits while supervising the work routines over several hours. The end-point for withdrawal from the chamber was impending collapse and always required careful judgement. Although the ethics of conducting such experiments in these post-war years were under consideration by the World Medical Assembly, the Declaration of Helsinki was not adopted until 1964. Apart from close physiological monitoring of the subjects, it was a generally accepted principle that we would not require volunteers to undertake anything we were not prepared to do ourselves.

One aspect which had not received systematic investigation at the time was the endocrine component of heat stress and adaptation reactions. In the Oxford department, we were able to undertake both animal and human experimentation and to take into account genetic variation. A colony of pure-bred mice had been reared for the purpose of genetic research, with F1 litters at birth being equally divided and reared in either hot or cool environments. Using radioactive iodine labelling, thyroid activity was shown to be depressed in heat-reared animals, and similar thyroid changes were then found in hot chamber studies on human subjects. In a later examination of the genetic influence, we performed thermoregulatory function tests on pairs of hetero- and homogenous twins using two thermal (Fox) beds operating in tandem. A major endocrine investigation was then planned to examine the role of adrenocorticosteroids, for which an experienced steroid chemist, J. D. Few, was appointed to develop the biochemical methodology. We were able to demonstrate the importance of glucocorticoids in the stress reaction [1] and the part played by the mineralocorticoid and aldosterone (known then as electrocortin) in controlling salt and water balance [2]. This included the first demonstration of the direct action of aldosterone on the eccrine sweat glands [3]. During the 10 years from 1954 to 1964 in Oxford, we had then established the endocrine basis of exposure to high environmental temperatures [4], and I completed a D. Phil as a member of Merton College with a thesis on the neuro-chemical control of the sudomotor system [5]. It was followed by a 3-month lecture tour of thermal laboratories in the USA during which I met most of the current authors of similar research work. On returning to Oxford, the head of Anatomy, Wilfrid le Gros Clark was about to retire, which meant that the MRC Unit had to be re-housed. Joe Weiner formed a new group entitled the MRC Environmental Physiology Unit located in the London School of Hygiene and Tropical Medicine (LSHTM).

## Clinical medicine and research

During the course of research in extreme environments, I had encountered a number of situations which had convinced me of the value and need for clinical training. Discussing this prospect with the MRC, Joe Weiner and my peers, I was encouraged to find total support. Finance was of course one of the main difficulties, but the MRC awarded me a Research Grant and Joe Weiner appointed me to a part-time post as scientific coordinator in the on-going International Biological Programme (1964–1974). As a result of the interest in human biology co-coordinating I. B. P. Human adaptability projects [6], I was assigned to be an editor of the journal* Annals of Human Biology*, which I held jointly with Geoff Harrison and Jim Tanner until 1994. Shortly before commencing the medical course at Guy’s Hospital, I was approached by the Chief Medical Officer of Esso Petroleum who asked whether I would lead a month’s expedition to study physiological changes and nutritional requirements of the crew of a modern tanker travelling to the Persian Gulf. There seemed to be some merit in this scheme which presented the opportunity of developing a number of new techniques for measuring body balances in a controlled environment. I was supported by a thermal technician, two nutritionist technicians and three medical students to act as control subjects on the 4-week heat acclimatization voyage to Bahrein. In three 3-day studies, the final one in the Gulf, total fluid, and nutrient balances were measured. All the samples were stored in deep freeze until the vessel later returned to the UK. The project was successful in identifying nutritional deficits associated with the hot environment and revealing many heat hazards in the working practices on board [7].

In the first year as a student at Guy’s Hospital, there was a requirement to qualify in anatomy and pharmacology before commencing the clinical course. Necessarily at that period, there was limited time to devote to MRC research with John Butterfield, who was the head of the medical department, but we managed to do so because of the advantage of being on site. The studies involved arteriovenous studies on the uptake and metabolism of cortisol in the muscles of the forearm in diabetic and other volunteers [8]. Qualification was followed by a year’s registration House jobs at Guy’s and the Royal Surrey County Hospital in Guildford, which is where my family and I lived. For some years, after that I commuted to London during the week days and became a Medical Officer in the Accident and Emergency Department at periods during the week-ends.

## Tropical medicine: Mecca and the Sudan

**Fig. 1:**
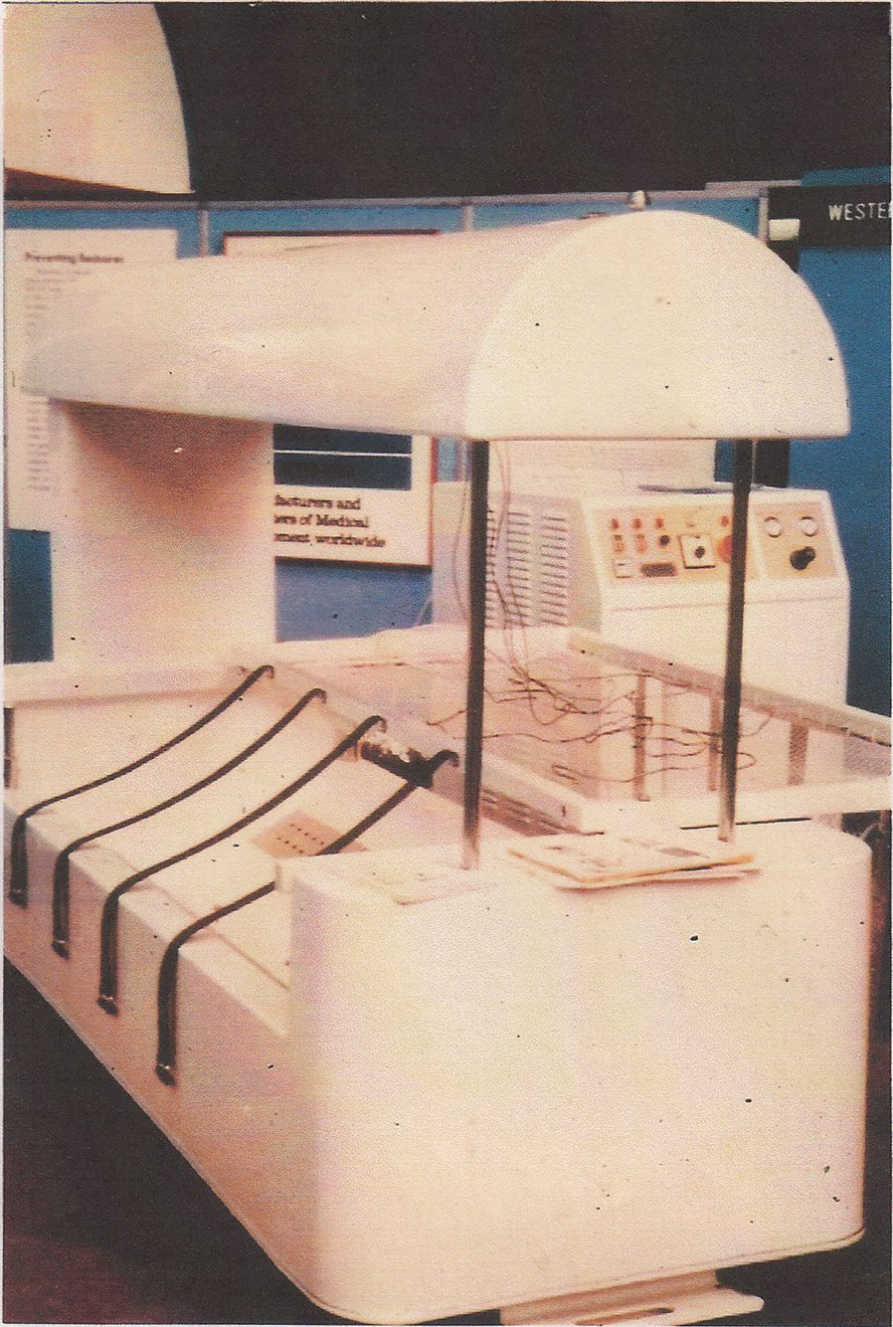
Mecca Body Cooling Unit for rapid cooling of hyperthermic and heat-stroke patients

On returning to the Unit at LSHTM, I found that Joe Weiner and Mustafa Khogali from Kuwait had constructed a Body Cooling Unit (BCU) with which to manage heat-stroke cases (Fig. 1), [9]. The purpose of the BCU was to achieve rapid cooling while avoiding peripheral vasoconstriction which inhibits the cooling process. With bursts of atomised water spray at 20 °C and high velocity warm air reaching the subject at 33 °C, skin temperature could be maintained above 31 °C as the body temperature is rapidly reduced. I supervised many of the initial tests on volunteer subjects which needed to be carefully controlled. The volunteers raised their deep body temperature to 39–40 °C by exercising on an ergometer in hot conditions while wearing an impermeable suit. Just before a heat-stroke level of body temperature was reached, they were transferred to the BCU after stripping off the suit and cooling commenced. After these tests had been satisfactorily completed, Khogali was able to transport the BCU technique to Mecca, so that it could be applied during the 7-day pilgrimage, the Mecca Hajj. With up to two million pilgrims attending annually, operating the facility along the pilgrimage route has been described as one of the largest human experiments in environmental physiology. Some thousands of people annually suffered heat illness and hundreds died of heat stroke before reaching treatment centres. These centres now possess BCUs which have the equivalent cooling power of approximately three times that of the average person sweating at their highest rate. Over the years, they have proved to be an effective life-saving procedure.

**Fig. 2:**
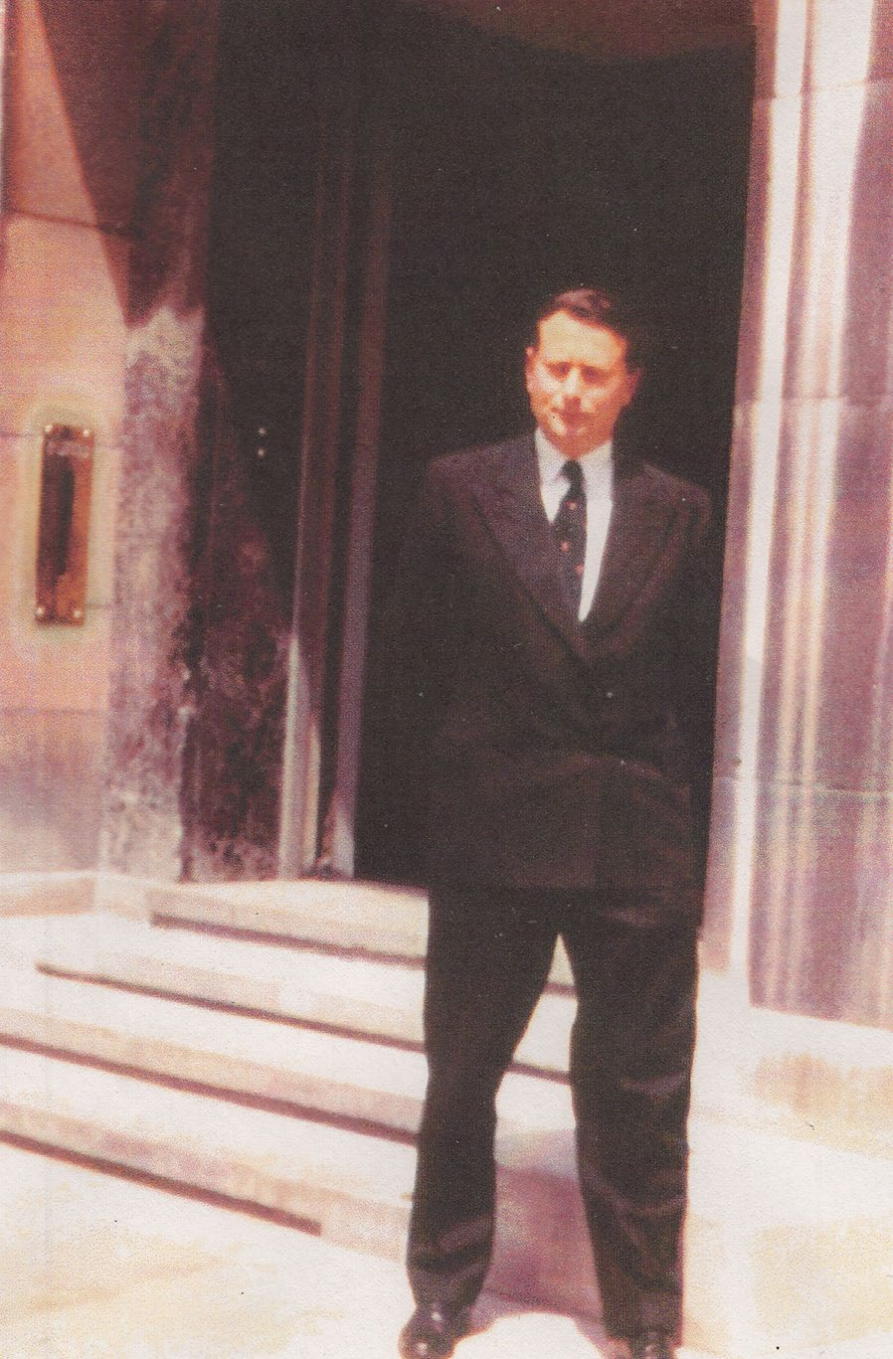
Author at the London School of Hygiene and Tropical Medicine. In 1974

At the LSHTM and the Hospital for Tropical Diseases, I became involved with the London–Khartoum Schistosomiasis Project (Fig. 2) which was designed to study and control the disease in the Gezira region of the Sudan, an important agricultural region situated between the two Niles. The Sudan Government wished to determine the possible reduction in working capacity of agricultural workers caused by the disease and the need for treatment. After a preliminary survey in the Sudan of the logistics and estimate of the resources required, I undertook the research which would occupy me by frequent working visits for the next 10 years. A little-used desert hotel owned by a sugar cane factory in the Gezira became the base for our accommodation, laboratory, and clinic. At first, the study population consisted of cane cutters living in the area who were infected because of a network of irrigation canals in the fields. We transported the men in groups of about 12 in the cooler early morning for aerobic working capacity measurements and screening tests for *S. mansoni*, malaria, and any other health problems. Analysis of samples was undertaken by the pathology department in Khartoum Hospital. We used hospital Identabands as a reliable identification method and these were prized like wrist watches by our volunteers. After the initial screening of about 400 cane cutters, we had the assistance of a number of excellent staff and medical students from Khartoum hospital to study the same cutters at work in the fields. They were set to work on designated areas with time scales and individual productivity recorded by conveying each man’s output to a weighbridge. Productivity could then be related to the severity of the disease measured by stool egg load [10]. From the first 3-month study, the statistical results on the field work surprisingly showed no clear disadvantage of a higher degree of *S. mansoni* infection, though the laboratory tests indicated otherwise. There was a missing factor, which we later found when analysing the skill of individual cutters. Most of those who were highly skilled at cutting had been doing so for many more years than newcomers and, therefore, had become more exposed to the disease. It appeared that the acquired skill had compensated for any physical detriment associated with the infection. Over succeeding years, clear evidence was found for the detrimental effect of the measured disease intensity on the performance of many other agricultural occupations [11]. When the infected volunteers had completed their participation, they were given treatment, and instructed on how to avoid re-infection. The other main aim of the project was to rid the canals of snails which are essential to the life cycle of schistosomes. This was achieved by spraying molluscicide onto the surface of the canals from aircraft. For the first 5 or 6 years, the snails appeared to have been eliminated from hundreds of miles of waterway, but after 10 years, there was evidence that the snails had begun to return to the areas first treated. The tropics were not easily going to give up one of its most elemental maladies.

## Urban hypothermia and thermoregulatory failure

I became interested in the thermoregulatory aspects and treatment of hypothermia after encountering cases in Guy’s Hospital, where surgery involving induced hypothermia was being explored. In the 1960s, the true incidence of hypothermia during cold winters in the UK population was unknown, and general practitioners reported thousands of cases in cold home surveys in S. W. England. I was introduced to Norman Exton-Smith the head of geriatric medicine at University College and St Pancras Hospitals who was keenly interested in the problem and provided a base to start investigations. During winter months, we surveyed about 2000 elderly people in their homes with the assistance of health workers to construct temperature profiles in the housing and their occupants. A longitudinal study of the age-related decline in thermoregulatory capacity was made on a random sample of 47 elderly people to try to identify those most at risk of spontaneous hypothermia. Although environmental temperature and socioeconomic conditions had not changed from 1971 to 1976, the core–shell temperature gradients were smaller in 1976, indicating progressive thermoregulatory impairment. People at risk of developing hypothermia had lower resting peripheral blood flows, a non-constrictor pattern of vasomotor response to cold, and a higher incidence of orthostatic hypotension [12]. In the population as a whole, about 5% of the elderly people had a lower than normal range of body temperature and only two people registered a uritemp below 35 °C. It appeared that the previous S. W. England survey had relied on oral measurements of deep body temperature which often provide inaccurate readings in cold environments. The MRC and the Department of Health invited me to discuss future work on the problem of hypothermia in elderly people.

**Fig. 3:**
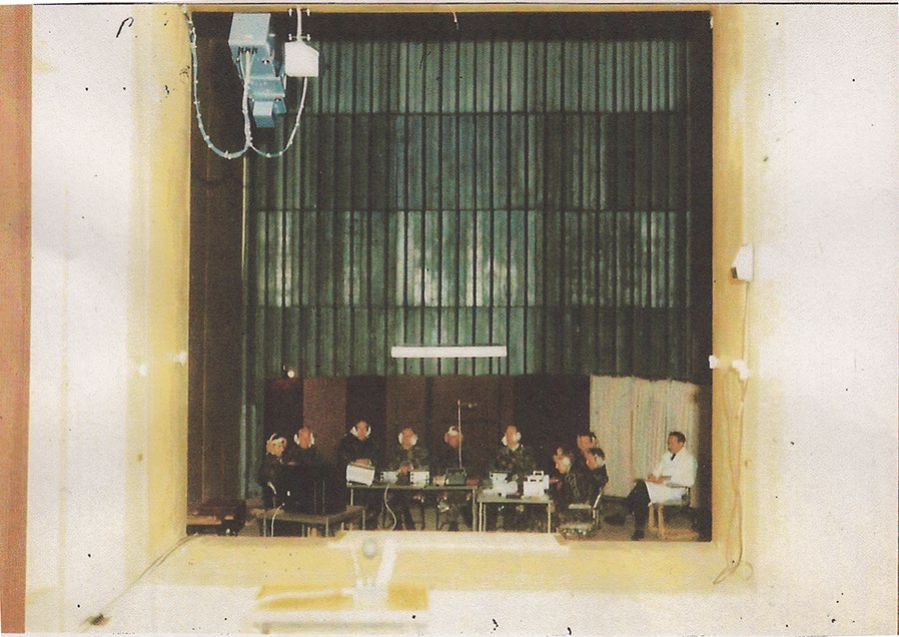
Group of veteran and young adult volunteers exposed to cold air conditions in an Army Personnel Research Establishment climate chamber

As a result of these discussions, it was decided that I direct a new MRC Unit at St Pancras Hospital to investigate the excess winter mortality. We developed methods to test elderly people on the three most important thermal physiological factors, thermogenesis, peripheral vasomotor responses, and central nervous control [13]. To measure the shivering response in elderly volunteers, the usual cold-water techniques were not to be contemplated, but I had at my disposable a Mecca BCU which provided a more acceptable approach using moving cool dry air. Shivering ability was not lost completely even in some of those over 80 years of age, but its character and peaks of power were changed. Investigations on groups of young and old adults were subsequently made with the generous co-operation of some of the UK. Energy companies and the armed services who provided the facilities of specialised climatic chambers (Fig. 3). It was then possible to make continuous 3-day studies of the diurnal rhythms of temperature, stress, and behaviour of young and older subjects living together in cool and cold environments [14]. With another facility, a self-controlled chamber fitted with a rapid temperature change ability, it was found that elderly people preferred the same mean comfort temperature, but manipulated ambient temperature swings much less precisely than the young [15]. In addition, although most young adults were able to discriminate by touch temperature differences of about 1 °C, elderly people could not match this, some failing to detect a 4 °C difference.

## Seasonal mortality and autonomic dysfunction

Although there are more deaths during cold winters in the UK, the evidence does not support the concept that hypothermia is the major cause of the well-recognised increase in seasonal mortality. An analysis by Graham Bull of daily deaths in England and Wales showed that mortality from heart attacks, strokes, and pneumonia increased linearly as environmental temperature fell from 20 to – 10 °C. In physiological investigations using thermoregulatory function test beds, we investigated the significant rise in blood pressure with cold conditions which were higher in elderly than younger volunteers [16]. We then showed that this was accentuated particularly in older people by facial cooling with cold air at 4 °C, while the body was kept warm. The trigeminal sensory reflex interactions were studied during breath-holding and with lower body negative pressure [17]. The direction of the Unit’s work changed following these studies, with more emphasis on clinical examination of patients with autonomic nervous system impairment [18]. The constraints of altered autonomic nerve function on the use of beta-blocking agents for treating hypertension in the elderly were investigated [19]. During this experimental work, close links were formed with the major centres of autonomic research at the Queen Square Hospital for Nervous Diseases and St Mary’s Hospital, London [20]. Our Unit studied postural hypotension on many referred patients using the lower body negative pressure technique, and in co-operation with the Royal Free Hospital in London, we commenced investigations on immunohistochemical changes in autonomic neuro-effector systems with ageing [21].

Other European countries faced similar problems with cold winter mortality to those in UK and I was appointed the UK permanent representative on the Comité de Direction of the Centre d’Etudes Bioclimatique in Strasbourg and became further involved in French biomedicine as a section editor of the* Journal de Physiologie*. This was followed by membership of European working groups for the World Health Organisation (WHO) on the health of older people in relation with indoor climate. In the 1990s, international concern had shifted from the effects of cold climates to that of heat in relation with potential climate change. Though the World Meteorological Organisation had at the time been unable to specify more precisely the likely extent of global increases in temperature the WHO convened the first Task Group in Geneva in 1990 to consider the potential health effects of climate change in which I participated as an adviser and member of the WHO Group. The report [22] outlined the mechanisms of climate change and its direct and indirect effects, most of which have not substantially altered during subsequent reports of the Group in later years.

## Comment

In the early phase of a research career, the satisfaction of being able to single-mindedly pursue a chosen path is difficult to surpass. Invariably that stage is succeeded by the addition of increasingly time-consuming professional obligations including teaching, supervision of higher degrees, clinical commitments and administration, and time-consuming but essential for gaining widening perspectives. Together with the constant support of a close family, the friendships which develop among colleagues working in collaboration or as part of a team are life-long and invaluable. It is often easy to overlook the fact that, especially in a field such as human applied physiology, there has always been a dependency on the co-operation of countless willing volunteers needed to make the experimental setting possible. I would like to express my gratitude to all of my past mentors and colleagues for their support and to Jo Marshall-Collins for assistance in the formation of this manuscript.

## Abbreviations

BCU: Body Cooling Unit
IBP: International Biological Programme
LSHTM: London School of Hygiene and Tropical Medicine
MRC: Medical Research Council
UCL: University College London
WHO: World Health Organisation
